# Early diastolic function observed in canine model of reperfused transmural myocardial infarction using high temporal resolution MR imaging

**DOI:** 10.1186/1532-429X-14-S1-W65

**Published:** 2012-02-01

**Authors:** Ziheng Zhang, Donald Dione, Ben A Lin, James S Duncan, Albert J Sinusas, Smita Sampath

**Affiliations:** 1 Diagnostic Radiology, Yale University, New Haven, CT, USA; 2 Cardiology, Yale University, New Haven, CT, USA

## Summary

We have applied a novel high temporal resolution MR imaging sequence to study diastolic function in canines with reperfused transmural infarction. Our results demonstrate abnormal diastolic strain-rates in infarct and viable risk region with corresponding abnormal filling patterns, as observed through the visualization of 2D flow pathlines 3 days post reperfusion.

## Background

Coronary angioplasty limits infarct expansion post myocardial infarction (MI). However, under certain conditions such as prolonged ischemia, the procedure induces reperfusion injury (RI), linked to adverse left ventricular (LV) remodeling and heart failure (HF). The functional mechanisms involved in adverse remodeling post reperfusion are still unclear. We have developed a new high temporal resolution MR imaging technique, SPAMM-PAV (SPAtially Modulated Magnetization with Polarity Alternated Velocity encoding) that provides regional assessment of early diastolic flow velocity and myocardial strain. This method was applied in a canine animal model with prolonged occlusion followed by reperfusion. We examine the diastolic strain-rates (index of stiffness) of infarct regions relative to remote regions and the 2D diastolic flow pathlines 3 days post reperfusion to provide insight into early diastolic function in these animals.

## Methods

**Figure 1:**
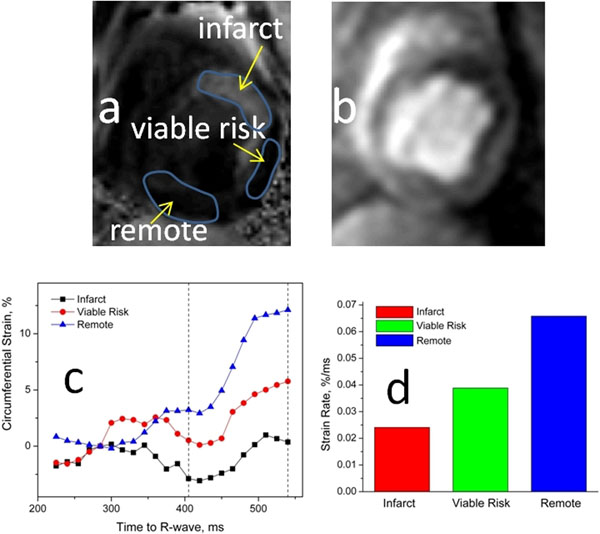
(a) Delayed hyper-enhanced image depicting infarct zone, and (b) corresponding first pass perfusion image depicting region of micro-vascular obstruction. (c) average diastolic strain curves and (d) average diastolic strain-rates in regions defined in infarct, viable risk and remote zones.

Studies were performed in six dogs following 5-6 hours of balloon occlusion of the left anterior descending artery (LAD) followed by reperfusion creating an antero-septal transmural infarct (see Fig. 1a) with reperfusion injury (see Fig. 1b). SPAMM-PAV measurements for both in-plane directions were conducted on a 1.5 T scanner on six short-axis slices and two long-axis slices, with imaging parameters set as follows: imaging matrix: 192×192, resolution: 1.5mm×1.5mm, slice thickness: 8mm, views per cardiac phase: 3, tag separation: 8mm, Venc: 120-150 cm/s, temporal resolution: 15-16ms. Late gadolinium enhancement imaging was performed to detect infarct region, and first pass perfusion imaging was performed to detect region of microvascular obstruction due to RI. Diastolic strain and strain-rates were computed from acquired SPAMM-PAV datasets using HARP analysis methods. For the long-axis slices, a set of blood emitter particles directly proportional to the area under the mitral inflow velocity curve were released from the valve plane at each time frame. The 2D flow velocity pathlines of each set of released emitter particles was tracked and superimposed. The pathlines were color-coded in correspondence to time of release.

## Results

**Figure 2:**
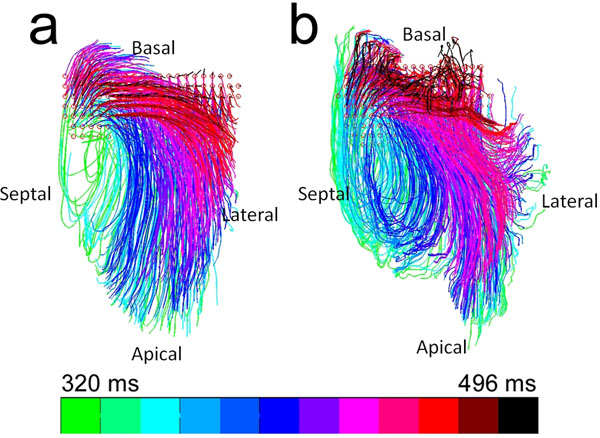
Overlapped blood emitter particle flow pathways for (a) normal canine and (b) canine with reperfused infarction (imaged 3 days post reperfusion), color-coded to indicate time of release of blood emitter particles.

The average circumferential diastolic strain and strain-rate in infarct region, adjacent viable risk region and remote region are shown in Figs. 1c and 1d. Note decreased diastolic strain-rates in infarct and viable risk regions, indicating increased stiffness in these regions. Fig. 2b. displays corresponding flow pathlines in a 4-chamber slice for the same animal. Note the abnormalities in the flow pathlines relative to a normal animal (Fig 2a), indicating dysfunctional filling in our canine model of reperfused transmural infarction.

## Conclusions

In conclusion, in a canine model of reperfused transmural infarction we observe decreased diastolic strain-rates in the infarct and viable risk regions relative to remote regions and corresponding abnormalities in diastolic filling patterns at 3 days post reperfusion.

